# Platelet functional alterations in a Bernard-Soulier syndrome patient with filamin A mutation

**DOI:** 10.1186/s13045-015-0171-z

**Published:** 2015-07-02

**Authors:** Jiaming Li, KeSheng Dai, Zhaoyue Wang, Lijuan Cao, Xia Bai, Changgeng Ruan

**Affiliations:** Jiangsu Institute of Hematology, The First Affiliated Hospital of Soochow University, Key Laboratory of Thrombosis and Hemostasis of Ministry of Health, 188 Shizi Street, Suzhou, 215006 China

**Keywords:** Bernard-Soulier syndrome, Platelets, Glycoprotein Ib-IX complex, Thrombocytopenia, Mutation

## Abstract

Defects in filamin A (FLNA) gene could lead to low platelet counts and decreased surface expression of glycoprotein (GP) Ibα. Here, we report and investigate the FLNA genomic alteration of a case with Bernard-Soulier syndrome (BSS), a rare hereditary bleeding disorder caused by quantitative or qualitative abnormalities in the GP Ib-IX-V receptor. DNA sequencing analysis reveals the presence of a GP Ibα c.987G > A mutation and a FLNA c.1582 G > A mutation in this patient. Transient transfection studies show that GP Ibα c.987G > A mutation abolishes the surface expression of GP Ibα on the transfected CHO cells. On the other hand, abnormal responses to collagen, including the platelet aggregation, secretion, and GP VI signaling pathways, are associated with FLNA c.1582G > A mutation. Our findings confirm a central role for FLNA in platelet-adhesive functions. The interaction between FLNA and GP Ibα in platelets deserves to be investigated.

## To the Editor

Filamin A (FLNA), a dimeric actin crosslinking protein, anchors the platelet adhesion glycoprotein (GP)Ib-IX-V receptor to actin cytoskeleton. The GP Ibα-FLNA interaction is essential for the platelet adhesion to von Willebrand factor that binds the GP Ib-IX-V receptor, for normal signal transduction reactions involved in platelet activation, and for maintaining normal platelet shape and integrity [1]. The absence of or mutations in the GP Ib-IX-V receptor or FLNA gene is responsible for Bernard-Soulier syndrome (BSS) or macrothrombocytopenia [2], respectively. We described here a female BSS case with FLNA mutation, showing her platelet functional defects in the platelet aggregation, secretion, and GP VI signaling pathways.

**Fig. 1:**
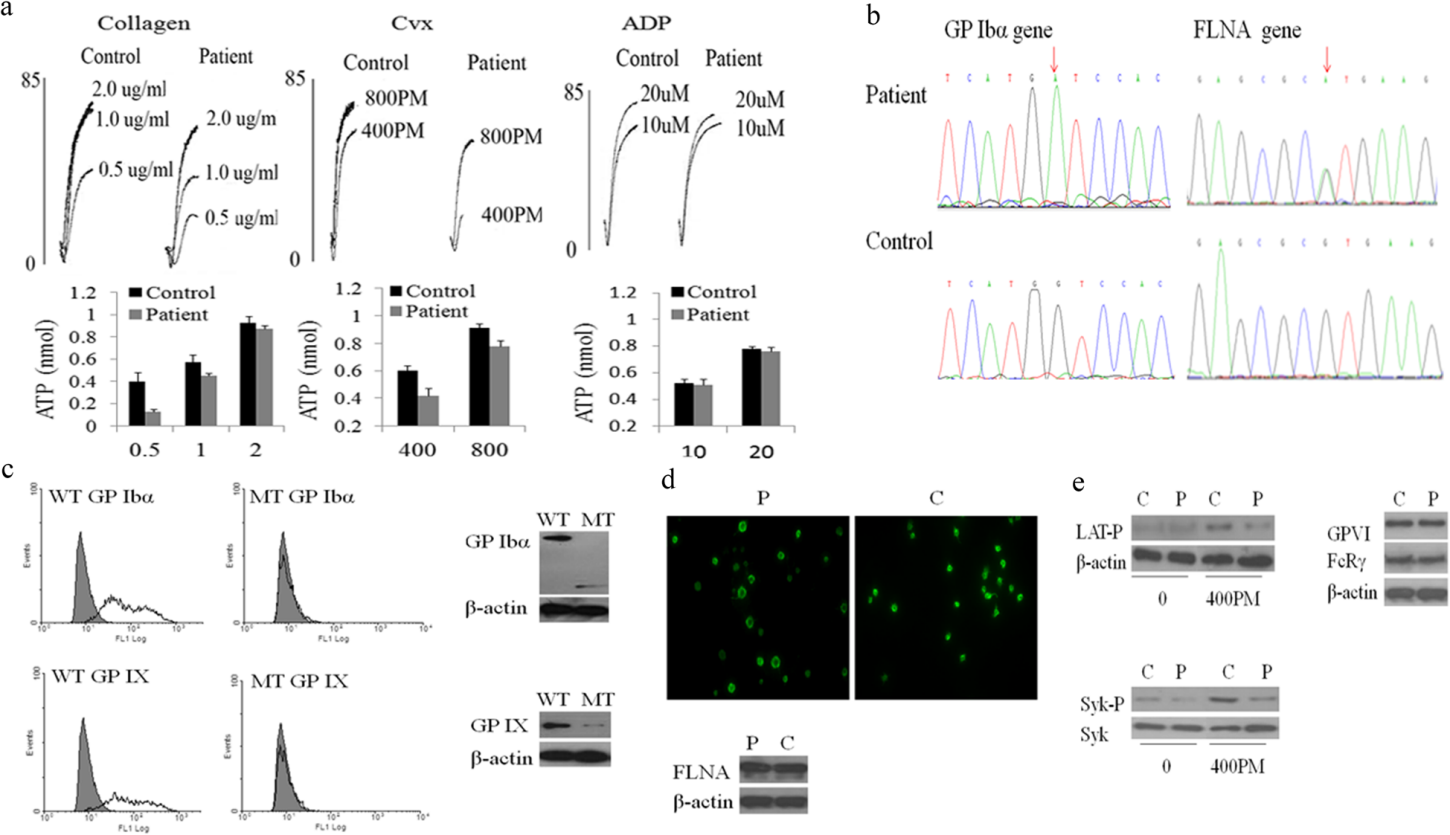
The effect of GP Ibα c.987G > A mutation and FLNA c.1582G > A mutation on the platelet functions. **a** Platelet aggregation and secretion induced by collagen, convulxin (Cvx) and ADP. Aggregations of patient were expressed as the percentage change in light transmission. The levels of ATP release were expressed as the amount of ATP released (nmol). Traces were representative of at least 2 experiments. **b** Part of the GP Ibα gene sequence with the c.987G > A mutation and FLNA gene sequence with the c.1582G > A mutation. **c** Flow cytometric analysis and immunoblotting analysis of GP Ibα and GPIX expression on transfected CHO cells. (*white curve*) CHO cells incubated with specific MoAb against GP Ibα (SZ2) or GPIX (SZ1), (*gray curve*) CHO cells with vectors pcDNA 3.1(−) incubated with mouse IgG. *WT* means wild-type GP Ibα; *MT* means mutant GP Ibα. **d** The level and distribution of FLNA using a monoclonal antibody specific for the C-terminal FLNA. Fluorescent microscopy showed that FLNA was mostly found in a peripheral layer, which was similar with that of normal controls. These results were representative of at least 2 independent experiments. **e** Platelet signaling induced by convulxin (Cvx).Washed platelets in suspension were activated by Cvx (400 PM) in the absence of stirring. Tyrosine phosphorylation of Syk (Syk-P) and LAT (LAT-P) was assessed by immunoblotting with an anti–Syk-P and anti–LAT-P, respectively. These results were representative of at least 3 independent experiments. *C* means control; *P* means patient

Patient’s platelet counts ranged between 20 and 30 × 10^9^/L, and enlarged platelets were observed in her blood film. Platelet aggregation on ristocetin was null. Collagen was decreased to induce platelet aggregation (0.5 μg/ml, 17 %; 1 μg/ml, 37 % versus 45 %, 70 % of controls). The GPVI-specific agonist, convulxin, was also decreased (400 PM, 10 %; 800 PM, 40 % versus 55 %, 70 % of controls). Platelets released 33–86 % less ATP than control platelets (Fig. 1a). The GP Ibα expression was practically undetectable on the platelet surface. DNA sequencing analysis revealed the presence of a new GP Ibα c.987G > A mutation in a homozygous state and a FLNA c.1582 G > A mutation in a heterozygous state (Fig. 1b).

To establish that GP Ibα c.987G > A mutation was responsible for the lack of GP Ibα surface expression in the patient’s platelets, we expressed in Chinese hamster ovary (CHO) cells the mutant or normal GP Ibα cDNA together with normal human cDNAs of GP Ibβ and GPIX. Only trace amounts of GP Ibα and GPIX was detected on CHO cells harboring the mutation, but truncated GP Ibα and normal GPIX was present in the CHO cell lysates (Fig. 1c). Together, these data could suggest shorten GP Ibα and GPIX were synthesized, but failed to be anchored and inserted into the plasma membrane.

A question raised by our observation was that patient’s platelet responses to collagen were impaired, despite a normal amount of platelet GPVI and FcRγ chain in her platelets. Several lines of evidence demonstrated that FLNA acted as a signaling scaffold for GPVI through interaction with tyrosine kinase Syk [3]. In our study, the normal level and regular distribution of FLNA was detected in the patient’s platelets (Fig. 1d). However, the phosphorylation of Syk was low (41 % of control) in the platelet signaling pathway of GPVI induced by Cvx (400 pmol/L). Similarly, the phosphorylation of LAT, a direct substrate of Syk, was decreased (45 % of control) (Fig. 1e). FLNA could bind to the tyrosine kinase Syk through its immunoglobulin-like repeat 3–5 in platelets [4]. In our patient, the identified FLNA c.1582G > A, an Ig repeat 3 mutation, interfered with the Ig repeats engaged in signaling of GPVI–collagen interaction. In turn, platelet aggregation and ATP secretion on collagen was interrupted. We thus conclude that abnormal responses to collagen were associated with FLNA c.1582G > A mutation.

To the best of our knowledge, this was the first report of a FLNA mutation causing abnormal response to collagen in a BSS patient. According to our results, we demonstrated that the synergistic effect of both GP Ibα c.987G > A mutation and FLNA c.1582G > A mutation could lead to the platelet functional alteration, including aggregation, secretion, and the GPVI signaling pathway. The relationship between GP Ibα and FLNA should constitute an area of interest for future studies.
